# The more the better? Effects of L1 tonal density and typology on the perception of non-native tones

**DOI:** 10.1371/journal.pone.0291828

**Published:** 2023-09-21

**Authors:** Min Zhu, Fei Chen, Xiaoxiang Chen, Yuxiao Yang

**Affiliations:** 1 School of Foreign Languages, Hunan University, Changsha, China; 2 Foreign Studies College, Hunan Normal University, Changsha, China; University of Nicosia, CYPRUS

## Abstract

This study investigates the effects of L1 tonal density and typology on naïve listeners’ perception of L2 Cantonese tones and pitch-equivalent pure tones. Native speakers of two canonical tone languages (Vietnamese and Mandarin) and a pitch-accent language (Japanese) with varying degrees of tonal density were recruited as listeners in a discrimination task followed by a perceptual assimilation task. Results implied that Mandarin listeners with a sparser tone inventory exhibited significantly better performance than Vietnamese listeners, suggesting that denser tonality in L1 did not facilitate or even interfere with L2 tone perception. Furthermore, both groups of canonical tone listeners processed pitch contours in a domain-general manner, with comparable performance in the perception of lexical tones and pure tones. However, Japanese listeners of the pitch-accent language perceived pure tones better than lexical tones, showing a domain-specific mechanism. These findings suggest that both L1 tonal density and typology may modulate the perception of non-native tones.

## Introduction

Tone languages (including pitch-accent languages) account for approximately 70% of the world’s languages and are spoken by more than half of the world’s population [1]. Non-native tone perception has been a topic of intense investigation for decades. It has been widely reported that second language (L2) learners often struggle to discern non-native tonal contrasts that are not present in their native language [2–4], even for those from a tonal language background [5–7]. So far, much attention has been paid to the contrast between tone language and non-tone language listeners [5, 6, 8–11]; In general, L1 tonal experience has been found to facilitate non-native tone perception, but in some cases, contradictory findings have been reported in specific tone languages [5, 6]. In addition to some extralinguistic factors like sample size and participants’ musical backgrounds, intrinsic factors like L1 tonal density might affect the degree to which tonal experience confers an advantage [5, 9, 12]; However, the examination of L1 tonal density in the context of non-native tone perception has been relatively limited. Therefore, this study was motivated to fill this gap by recruiting listeners from Vietnamese, Mandarin, and Japanese backgrounds, which differ a lot in the number of tones.

Regarding L1 tonal typology, tone languages could be further split into canonical tone languages (e.g., Mandarin, Vietnamese, and Thai), and pitch-accent languages (e.g., Japanese, Swedish) based on pitch realizations [1]. In canonical tone languages, pitch variations occur on individual syllables, while in pitch-accent languages, pitch varies across consecutive syllables rather than individual ones [11]. Moreover, instead of the absolute dominant status of lexical tones in canonical tone languages, pitch is realized restrictively in pitch-accent languages, with sparse distribution on some words [13, 14]. Extant research has shown that pitch processing mechanisms in speech and non-speech contexts are subject to L1 typology [15–17]. For tone languages, it was found that Mandarin and Cantonese listeners perceived tones categorically in both speech and non-speech contexts, implying that pitch processing ability in a tone language could be transferred to the non-speech domain [16, 17]. Non-tone language listeners, on the other hand, showed increased sensitivity in the non-speech context compared to the speech context, indicating unequal pitch processing capacities across domains [15, 16]. However, it is still unclear whether the mechanisms underlying tone perception in linguistic versus non-linguistic contexts differ as a function of L1 tonal typology. To this end, the present study aimed to explore how experience with canonical tone and pitch-accent languages influenced naïve listeners’ perception of Cantonese tones (CT) and pitch-equivalent pure tones.

Furthermore, L1 tonal typology has also been shown to affect listeners’ perceptual bias towards different acoustic cues in the perception of tones [2, 18, 19]. It was found that Mandarin and Cantonese listeners paid more attention to the pitch contour, while Japanese listeners attended more to the pitch height [19]. To the best of our knowledge, Vietnamese is one of the canonical tone languages that has been less studied than others. This makes it unclear how Vietnamese listeners perceive non-native tones and the relative importance of pitch height and contour in their perception.

Concerning tone perception, it is widely recognized that listeners’ discriminability for non-native tones relies upon the cross-language perceptual similarity between the target language and their native language [4, 11], which is frequently assessed through a perceptual assimilation task. Accordingly, the Perceptual Assimilation Model (PAM) [20], including its expanded version, PAM for suprasegmental (PAM-s) [4], proposed six assimilation patterns to predict listeners’ degree of success in discriminating non-native sounds. If listeners could consistently map a non-native contrast onto two categories (*Two Category*; TC) of their native language, the discrimination performance would be excellent, whereas the worst discrimination occurs for the pattern of *Single Category* (SC) or *Category Goodness* (CG), under which pairs are categorized into one native category. As the assimilation pattern is closely correlated with discrimination results, it is therefore of interest to examine how patterns of assimilation vary as a function of L1 tonal density. The Second Language Linguistic Perception Model (L2LP) [21] posited that perceptual acquisition would be considerably easier when the size of the L1 phonological system was no less than the target language. Based on the above, this study would explore the effects of L1 tonal density on listeners’ assimilation and discrimination of non-native sounds. Expectations may arise that listeners with a denser tone system will find it easier to assimilate non-native tones into two categories, which in turn enhances their discrimination accuracy. Before going into depth about our research, previous literature regarding the L1 inventory and speech vs. non-speech processing will be systematically reviewed to elucidate the motivation of the present study.

### Studies concerning L1 inventory

L1 inventory size has been found to affect L2 speech perception, with relevant research mainly focused on the segmental level [22–26]. For example, it has been found that listeners’ degree of success in perceiving L2 vowels is closely related to the number of vowels in their native language [22, 25]. Specifically, when the learners’ L1 vowel system is smaller than the L2 system, many L2 contrasts tend to be perceived as single native categories, leading to considerable confusion in discrimination [26]. As such, it was reported that German listeners performed worse than their Danish counterparts at discriminating English approximant contrasts owing to a smaller inventory of approximants in the German system compared to the Danish system [22].

However, compared with abundant research at the segmental level, there is less empirical research regarding the effect of L1 inventory size at the suprasegmental level [6, 9, 12]. A pioneer research [9] investigated Mandarin, Cantonese, and English listeners’ discrimination of Mandarin and Cantonese tonal contrasts. The study found that Cantonese listeners outperformed their English counterparts in perceiving Mandarin tones; However, such superiority was not observed in Mandarin listeners’ perception of Cantonese tones. The authors attributed the asymmetry of the results to the disparity between Cantonese and Mandarin tone inventories, with the former being denser than the latter. In other words, it is likely that tone language experience in L1 could be positively transferred to L2 only when L1 is more complex than L2. Similarly, the facilitating effect of a denser L1 tone system is also upheld by [12], in which Cantonese listeners showed greater sensitivity to phonetic distinctions of tones compared to Mandarin listeners. Some other studies, however, have reported an opposite effect, namely that the presence of a denser tone language would not necessarily foster the perception of non-native tones. One study [6] investigated how three populations with various prosodic features, Hmong, Japanese, and English, perceived Mandarin tones. It was revealed that native speakers of Hmong, a language encompassing seven lexical tones, performed the worst among all groups at the initial stage. Likewise, another study [11] found that Cantonese listeners performed worse than Japanese and English listeners in discriminating some Mandarin tone pairs despite a denser system in Cantonese relative to Japanese.

To conclude, despite disputes in previous studies, the size of the native tone inventory seems to play a role in the transfer of L1 tonal experience to L2. This could potentially provide a plausible explanation for ambiguous tonal experience in tonal vs. non-tonal comparison. However, there were some confounding issues in previous studies that might affect their conclusions. Firstly, there was no restrict control of participants’ music experience in the [9], which has been proven to affect pitch sensitivity [27]. Secondly, mixed conclusions could stem from different comparison criteria used in previous studies. For instance, while some studies focused on listeners’ overall performance [6, 9], others drew conclusions based on specific tone pairs [11, 12]. Thirdly, although some of the previous studies involved listeners from different tone languages, they mostly concentrated on the comparison between tone language and non-tone language listeners, and could not thoroughly explore the impact of tonal density by comparing two canonical tonal L1s [6, 9, 11]. For instance, there was no direct comparison between Cantonese and Mandarin native listeners in their perception of a third language [9, 12]. Hence, a lack of comprehensive comparisons across diverse tone languages in the above studies may suggest the need for further research involving more L1 tonal systems.

### Studies concerning the perception of speech and non-speech tones

A number of studies have examined the perception of speech and non-speech tones with respect to the issue of domain-generality vs. domain-specificity [15, 17, 28–33]. Two different views exist regarding how speech and non-speech tones are processed. Some studies [29, 31] argued that musical tones and lexical tones were processed by a general mechanism in the brain, whereas [32] underpinned the proposal of a speech-specific mechanism. They found that Cantonese listeners outperformed the tone-merging individuals for lexical tones, yet showed no advantages for musical tones when lexical information was removed, which indicates a more fundamental role for lexical tones than non-speech tones [32].

In addition, some evidence suggests that whether speech and non-speech tones share a unified processing mechanism is determined by the typology of the listener’s L1 [15, 17, 34–36]. For instance, asymmetry was observed in the discrimination of speech vs. non-speech tones by tone and non-tone language speakers, which might originate from the perceptual reorganization of lexical tones occurring in infancy. One study [35] demonstrated that although infants born into a non-tone language environment failed to detect tonal distinctions in the linguistic context between six and nine months, there was no parallel deterioration in non-speech tonal discrimination. Results indicated that the absence of lexical tones in the phonological system precluded non-tone listeners from discerning lexical tonal contrasts but not musical tones. Moreover, a relevant study [15] extended the findings to adults by finding that English speakers showed significantly higher performance when the stimuli became less speech-like. In contrast, Mandarin and Cantonese listeners exhibited comparable accuracy and neural responses (MMN) for both speech and non-speech tonal stimuli [15, 34–36], supporting the idea of a shared pitch processing mechanism across domains for canonical tone languages.

In addition to a broader typology, pitch processing might be affected by subtypes of tone languages. There is evidence that listeners of canonical tonal languages integrate syllable and tone perception, whereas pitch-accent language listeners process segmental information and pitch variations independently [19]. Given this language-specific processing feature, it raises the question of whether and how pitch processing mechanisms may differ between speech and non-speech domains for canonical tone and pitch accent L1s. Most previous studies either focused on comparisons between tone and non-tone language listeners [15, 17, 33, 35] or recruited listeners from a single language background [28, 30, 31], limiting our understanding of cross-language differences in pitch processing mechanisms. Therefore, the study seeks to explore how L1 tonal typology modulates non-native tone perception in speech and non-speech contexts.

### The present study

Given the research gaps mentioned above, the present study explores Cantonese tone (CT) perception in both speech and non-speech contexts by Vietnamese (high tonal density), Mandarin (medium tonal density), and Japanese (low tonal density) listeners. It would be meaningful to investigate whether Vietnamese listeners with a larger tone inventory have an advantage over Mandarin and Japanese listeners in processing Cantonese tones by virtue of potentially more TC mappings in assimilation. Moreover, this study aims to address the question of whether there is a shared general mechanism for processing speech and pitch-equivalent pure tones across different types of tone languages, such as canonical tone languages and pitch-accent languages.

Cantonese was chosen as the target language because it has a large inventory size (six contrastive tones) and a balanced tonal type, comprising three level tones: CT1 [high-level tone], CT3 [mid-level tone], and CT6 [low-level tone], and three contour tones: CT2 [high-rising tone], CT4 [low-falling tone], and CT5 [low-rising tone]. As such, it would not induce any bias for listeners with different cue weightings and effectively avoid the ceiling effect. In Cantonese, the six contrastive tones are distributed unevenly in the acoustic space, with CT1 being at the top of the space and the other five tones being crowded below the space [37]. Due to intrinsic phonetic similarity, three level-tonal contrasts (CT1-CT3, CT3-CT6, CT1-CT6) and CT2-CT5, CT4-CT6 have been reported to be perceptually ambiguous even for native speakers when surrounding context information is not available [38, 39].

**Fig 1:**
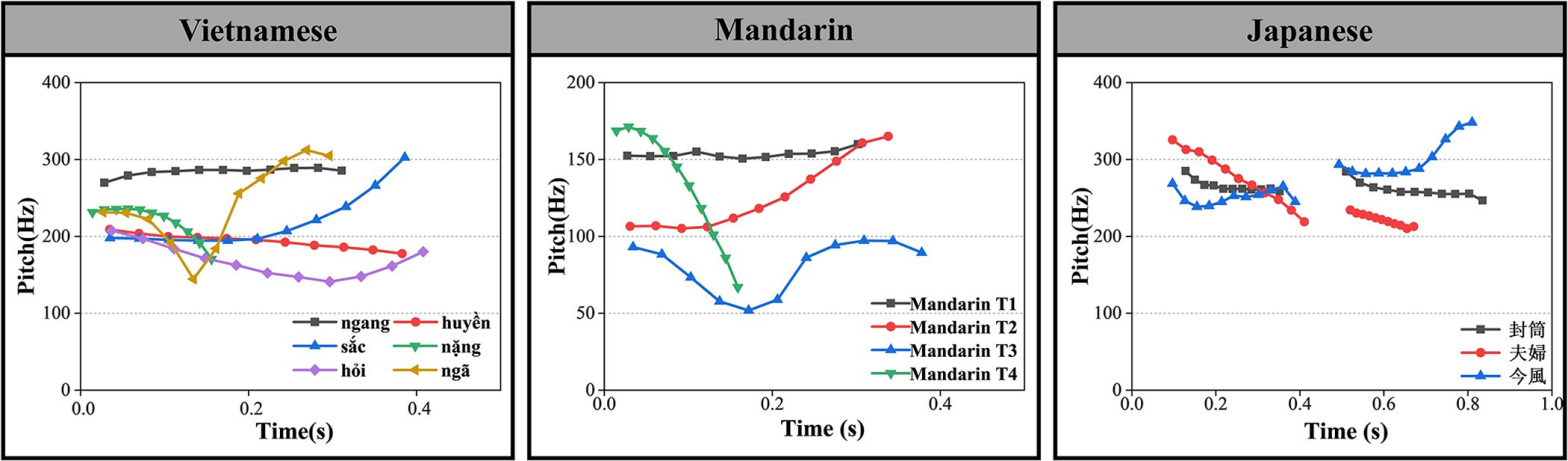
F0 patterns of Vietnamese tones (left panel), Mandarin tones (mid panel), and Japanese pitch accents (right panel). The Vietnamese and Mandarin tones were carried by the syllable /ma/; the Japanese pitch accents were carried by the word /fu:/ adapted from [40].

As a representative of canonical tone languages, Mandarin encompasses four lexical tones (Fig 1): Tone 1 (high-level tone); Tone 2 (high-rising tone); Tone 3 (dipping tone); Tone 4 (high-falling tone). Each of the Mandarin tones is carried by a monosyllable. Despite the presence of other cues such as duration and intensity, pitch serves as the primary cue in Mandarin to differentiate lexical meanings [18]. In addition, since Mandarin tones are principally defined by pitch contour [37], it has been reported that Mandarin listeners are more sensitive to pitch contour than pitch height in tone perception [19, 39].

Likewise, as shown in Fig 1, Vietnamese is also a canonical tone language, but with a larger tone inventory size. Additionally, unlike Mandarin, the six contrastive lexical tones in Vietnamese are characterized not only by pitch contours but also by voice quality [41, 42]. More specifically, the tone ngang “level” exhibits a flat contour, while the tone sắc “sharp” is a rising tone. The tone huyền “deep” starts relatively low and falls smoothly. The tone nặng “heavy” is also a falling tone but is typically shorter, with a glottal stop. The tone ngã “tumbling” is a falling-rising tone, which is interrupted by a creaky voice. The tone hỏi “asking” falls dramatically until it reaches a turning point with a slight laryngealization. However, in colloquial Hanoi speech, the tone hỏi has lost its final rise [41].

Japanese is a pitch-accent language, which is considered a subtype of tone language [1, 14]. Although pitch variations are used to contrast lexical meanings at the lexical level, they are realized differently in Japanese. Specifically, Japanese uses pitch variations restrictively, over two timing units (moras) rather than on a single syllable (あめ/ame/: rain and malt). In addition, unlike canonical tone languages, pitch variations in Japanese are minimal or completely absent for certain words. Therefore, Japanese is often referred to as a typological intermediary between tone and non-tone languages [43]. Japanese pitch variations are realized in three patterns, as shown in Fig 1: “high-high”, “high-low”, and “low-high”. Taking /fu:/ as an example, three pitch-accent patterns correspond to “封筒/fu: toː/”, “夫婦/fuː fu/”, and “今風/ima fuː/”, respectively.

It is noteworthy that the level of gradient in different tonal systems of Vietnamese, Mandarin, and Japanese provides an ideal window for investigating the effect of tonal density. Specifically, by conducting perceptual assimilation and discrimination tasks, this study aims to address the following three questions:

RQ1: How does the size of the L1 tonal inventory affect naïve listeners’ assimilation of non-native Cantonese tones?
RQ2: How does the size of the L1 tonal inventory affect naïve listeners’ discrimination of non-native Cantonese tones?
RQ3: How does L1 tonal typology shape pitch processing across different domains?

## Materials and methods

### Participants

A total of 70 young college students participated in the present study, consisting of 20 native Mandarin speakers (10 males and 10 females; Mean age = 22.65 years, SD = 2.31), 15 native Japanese speakers (7 males and 8 females; Mean age = 20.87 years, SD = 1.50), 15 native Vietnamese speakers (7 males and 8 females; Mean age = 23.87 years, SD = 2.56) and 20 native Cantonese speakers as a control group (10 males and 10 females; Mean age = 20.60 years, SD = 1.74). All participants self-reported no history of speaking, hearing, or cognitive disabilities. According to the questionnaires, the three experimental groups had no prior knowledge of Cantonese or formal musical training outside the classroom, which could facilitate listeners in discerning phonetic distinctions [27].

Native Mandarin speakers were born and raised in Northern China. They spoke standard Mandarin without any other dialects. Native Japanese and Vietnamese speakers were exchange students in Changsha, residing in China for less than half a year, and they had never been exposed to other tone languages in their countries. The control group, Cantonese speakers, were natives of Guangdong province. Before entering college, they spoke Cantonese in daily life. In addition, their Cantonese proficiency was verified by another four native Cantonese elders through the reading of *The North Wind and the Sun* [44].

Before the experimental tasks, they had all passed a pure-tone hearing screening (250–8000 Hz at 25 dB hearing level). Prior to the experiment, approval of the research was granted by Human Research Ethics Committee of Hunan University. Written informed consent was obtained from all participants in compliance with the experiment protocols. Participants were recruited from September to December 2019, and they were financially compensated for their participation.

### Materials

Stimuli included two types, monosyllabic real words, and homologous pure tones. Two target syllables /ji/ and /fu/ as well as fillers /se/ and /jɐu/ with six CTs were embedded in a carrier phrase context: ŋɔ kɔŋ x (“I say x”) [11]. The recording word list is shown in Table 1. Stimuli were recorded five times by two native Cantonese speakers (a female and a male, Mean age = 20.00 years) from Guangzhou in a sound-attenuated room under the experimenter’s supervision. All the recordings were conducted individually via a professional microphone (Shure Beta 58a, Niles, IL) linked to an external sound card (Avid Mbox 3, Burlington, MA) at a sampling rate of 44.1 kHz with 16-bit resolution. Speakers were advised to speak Cantonese for five minutes before recording to accommodate language switching. Then they read carrier sentences presented randomly on the computer screen at a natural speed, yielding a total of 240 sentences (4 syllables × 6 tones × 5 repetitions × 2 speakers).

**Table 1:** The wordlist of target syllables (/ji/ and /fu/) and fillers (/se/ and /jɐu/) carrying six tones.

	CT1 (55)	CT2 (25)	CT3 (33)	CT4 (21)	CT5 (23)	CT6 (22)
/ji/	醫 to cure	椅 chair	意 meaning	兒 son	耳 ear	二 two
/fu/	夫 husband	斧 axe	富 rich	符 symbol	婦 woman	父 father
/se/	些 some	寫 write	瀉 spill	蛇 snake	社 society	射 shoot
/jɐu/	休 rest	柚grapefruit	幼 young	油 oil	友 friend	右 right

*Notes*. CT = Cantonese tone. Numbers in brackets represent the “five-degree” pitch values of each tone.

Syllables /ji/and /fu/ were used as target syllables since they could be affixed to any of the six CTs to form real words in Cantonese and were adopted in the previous study [2]. In addition, /ji/ and /fu/ had similar counterparts in all relevant languages to minimize the confounding effect of unfamiliar segments on tone perception [19].

All target words were extracted and checked for spectrogram and waveform via Praat [45]. Two tokens per word were finally chosen based on similar duration and clear F0 curve. All tokens were confirmed to be intelligible by the four native Cantonese elders mentioned above. To generate the non-linguistic/non-speech pitch counterparts, F0 trajectories of six CTs were first extracted from the zero-onset syllable /ji/. Then, six pure tones were generated and replaced with the pitch tiers extracted from the syllable /ji/ via the pitch-synchronous overlap-and-add (PSOLA) operation in Praat. Finally, all stimuli (both speech and non-speech) were normalized to 75 dB intensity and 600 ms duration to avoid the effect of duration and intensity on tone perception.

### Procedures

The whole experiment consisted of two sessions, including a two-alternative forced-choice discrimination task performed by all groups, and a perceptual assimilation task administered solely by the Vietnamese and Mandarin groups. The Japanese group was excluded from the assimilation task since Japanese speakers lacked overt tonal categories and failed to consistently establish the mappings of pitch patterns between Japanese and Cantonese in the practice phrase. All tasks were conducted independently by listeners via a laptop and a head-mounted microphone using the presentation program of Experiment MFC 7 from Praat in a quiet classroom. Task instructions were translated into their native languages: Vietnamese, Chinese, and Japanese for each corresponding group. Participants were instructed to complete the discrimination task first since assimilated responses would influence their discrimination judgments [4]. They were told that all sounds they would hear were from an unfamiliar language, and they would not receive any feedback during the 30-minute experiment. They could take a break between blocks as they intended.

#### AX discrimination task

A total of 432 trials were divided into six blocks (i.e., 72 trials per block) by stimulus (/ji/, /fu/, non-speech), and speaker (male, female). Each block was made up of 15 different pairs and 6 same pairs in four formats: AA, AB, BB, and BA (A and B represent different tones) repeated twice, resulting in a total of 60 different pairs and 12 same pairs per block. The task of speech discrimination was always prioritized over pure tone discrimination. Within each stimulus type, the presenting order of blocks was counterbalanced across the participants. For each trial, listeners could hear two sounds successively with an inter-stimulus interval (ISI) of 500 ms. These two sounds were identical in segments but carried the same or different tones, and participants were required to indicate whether the two sounds were the same or not by clicking the box labeled “same” or “different” on the laptop screen. It is worth mentioning that the two speech stimuli used in the “same” pairs were not the same acoustically (two tokens of one word), so listeners must make a decision based on “words” rather than “sounds”. All stimuli were randomly presented through the command “Permute Balanced”. Once participants made the response, the next trial would appear automatically 500 ms later. Before the formal test, each participant completed a series of practice trials using fillers.

#### Perceptual assimilation task

A perceptual assimilation task was designed to see how Vietnamese and Mandarin listeners assimilated Cantonese tones into their native tonal categories. There were 48 tokens (2 speakers × 2 tokens × 2 syllables × 6 tones) in this session, which were classified into two blocks by syllable. Pure tones were excluded from the assimilation task owing to a lack of linguistic significance. The stimuli were played randomly within each block. Participants were requested to choose a native counterpart that was most similar to the Cantonese tone they heard. The screen displayed the corresponding native tonal categories of each language (e.g., Tone 1 for Mandarin; ngang for Vietnamese) along with a “none” button if listeners could not assimilate the sound to any of the native tonal categories. After they selected the tonal category, they were required to rate the similarity between the tone they heard and the corresponding native category based on a 7-point Likert scale (1 represents “least similar” while 7 represents “very similar”). Listeners could listen to the sounds multiple times by clicking the “replay” button to ensure a confident response. After rating, a new trial would appear automatically. The rating score would be discarded if the listener picked “none” in the assimilation stage. Similarly, a familiarization block with 12 samples using fillers was performed before the formal task.

### Data analysis

#### AX discrimination task

Listeners’ sensitivity to correctly discriminate each trial was evaluated using the *d’* score [46] with log-linear adjustment available in the software “SDT Assistant V.1.0” (https://hautus.org/). The *d’* score serves to remove any response bias from sensitivity and has been widely adopted in tonal discrimination tasks [28, 47–49]. For model fitting purposes, 15 Cantonese tonal contrasts were separated into two dimensions: contrast by pitch height (T1-T3, T1-T6, T2-T5, T3-T6), and contrast by pitch contour (T1-T2, T1-T4, T1-T5, T2-T3, T2-T4, T2-T6, T3-T4, T3-T5, T4-T5, T4-T6, T5-T6). Such classification aims to compare listeners’ cue weighting during the pitch discrimination.

#### Perceptual assimilation task

Following previous studies [19, 50, 51], results of the perceptual assimilation task were measured by three criteria: first, assimilation percentages and similarity scores; then, the fix index and degree of response diversity (*K′*) as in [50]. As a metric, the fit index takes response rates and similarity ratings into account by multiplying the mean percentage of responses and the mean similarity rating scores. It is used to estimate the assimilation fit of an L2 category to L1 categories, and a larger fit index indicates a smaller perceptual gap between them. Moreover, another index, *K′*, was computed using the formula below:

$$K^{\prime }=\frac{1}{\sum_{i=1}^{R}P_{i}^{2}}$$ (1)

where R is the total number of L1 tonal categories, and Pi refers to the proportion of responses in which a non-native tone is assimilated into a specific L1 tone category. The lowest diversity (*K′* = 1) implies that a non-native tone category has been steadily assimilated into a single L1 tone category, whereas the highest diversity (*K′* = the number of L1 tonal categories) indicates that a non-native tone category has been discretely mapped onto all given choices in an unbiased manner. The degree of response diversity could reflect the assimilation consistency of each L2 tone category being mapped onto L1 tonal categories. Both fit index and *K′* values serve as useful parameters in revealing the degree of perceptual similarity between two phonetic inventories.

## Results

### Perceptual assimilation task

Listeners’ performance in the perceptual assimilation task would be first reported, since it forms the basis for predictions of discrimination sensitivities.

#### Assimilation percentages and similarity scores

**Fig 2:**
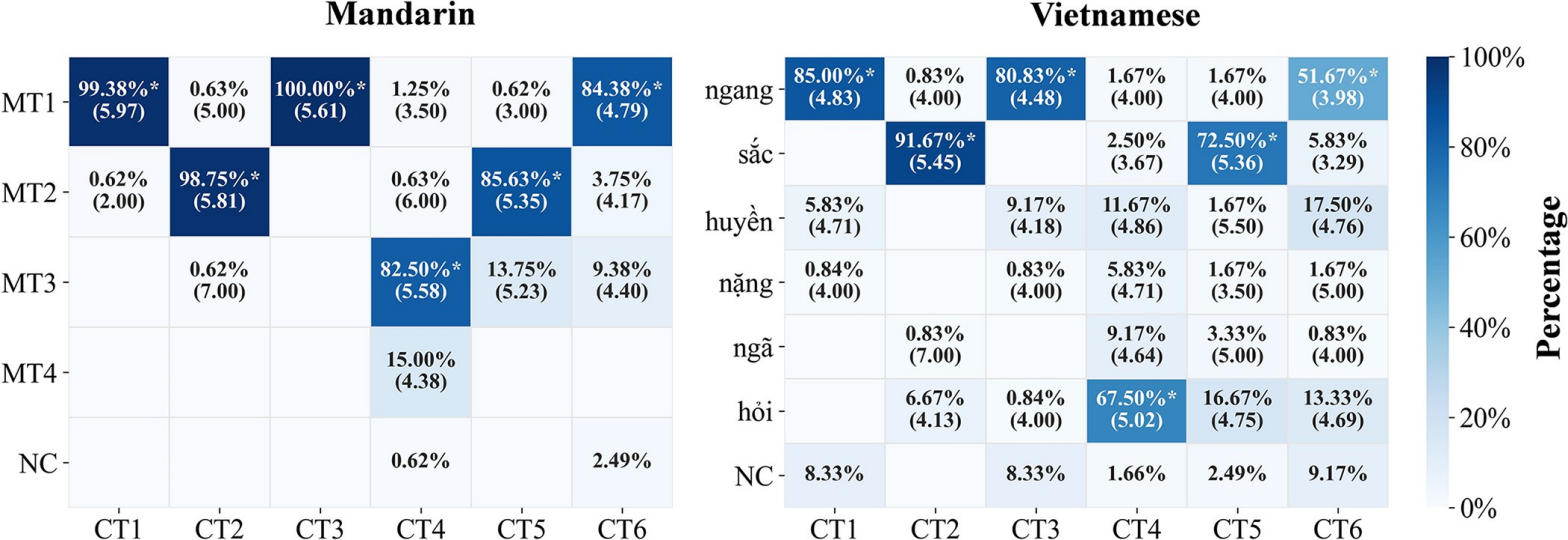
Assimilation matrix of Vietnamese and Mandarin categories for each Cantonese tone, with similarity ratings in brackets. Asterisks* refer to assimilated tones. The colors of the rectangles indicate assimilation percentages. NC = no category. MT = Mandarin tone. ngang = “level” tone. sắc = “sharp” tone. huyền = “deep” tone. nặng = “heavy” tone. ngã = “tumbling” tone. hỏi = “asking” tone.

Vietnamese and Mandarin listeners’ assimilation patterns between Cantonese and their native languages are depicted in Fig 2. In line with [4], one tone is considered assimilated only when its frequency is significantly higher than both the chance level and that of any other choice. Hence, in the present study, the chance level was about 14.29% for Vietnamese (seven choices, including one “none” option) and 20% for Mandarin (five choices, including one “none” option). For the statistical analysis, the assimilation percentages of Cantonese tones for both groups were analyzed using Generalized Linear Mixed Effect models (GLMMs) in R 3.6.1 [52]. Through the package *mlogit* [53], the multi-level categorical variables of Vietnamese and Mandarin options were converted into binomial distribution: “1” (the specific tone category was chosen) and “0” (the specific tone category was not chosen) as dependent variables, with “Vietnamese option” or “Mandarin option” as the fixed effect. “Subject” and “Item” were fitted as random factors. Main effects and interactions were assessed via the package *car* [54], with pairwise comparisons being performed through the package *emmeans* [55]. The statistical results for each language group would be reported separately.

*Vietnamese group*. Results showed a significant main effect of the “Vietnamese option” on all Cantonese tones (*p*s < .001). Further post-hoc pairwise analyses suggested that the frequency of the following Cantonese-to-Vietnamese assimilations was significantly above both the chance level (14.29%) and others (*p*s < .001): CT1 to ngang (85.00%); CT2 to sắc (91.67%); CT3 to ngang (80.83%); CT4 to hỏi (67.50%); CT5 to sắc (72.50%), and CT6 to ngang (51.67%), indicative of their corresponding assimilation patterns. Hence, all Cantonese tones could be assimilated into the Vietnamese tonal system based on the assimilation criteria. Specifically, CT1, CT3, and CT6 were assimilated to ngang, CT2 and CT5 were assimilated to sắc, and CT4 to hỏi. Furthermore, when two sounds are assimilated into a single native category, the similarity ratings need to be compared to determine whether it is SC or CG. Hence, listeners’ similarity rating scores were analyzed via Linear Mixed Effects Models (LMMs) to determine the specific assimilation type due to overlapping assimilations for Vietnamese tones ngang and sắc. In this model, “Similarity ratings” and “Cantonese tones” were incorporated as the dependent variable and fixed effect, respectively. Additionally, “Subject” and “Item” were calculated as random effects after model comparisons via the “anova” function in the *lme4* package. The visual inspection of Q-Q plots and plots of residuals revealed no obvious deviations from homoskedasticity. For ngang, results suggested that CT1 had a significantly higher similarity score than CT6 [*β* = 1.13, SE = 0.18, *t* = 6.43, *p* < .01], and a similar case was found with CT3-CT6 [*β* = 0.76, SE = 0.18, *t* = 4.35, *p* < .05]; however, similarity rating scores on CT1 and CT3 did not differ from each other [*β* = 0.36, SE = 0.15, *t* = 2.38, *p* = 0.22]. Similarly, no significant discrepancy was observed between CT2 and CT5 in terms of the similarity ratings on sắc. Therefore, for Vietnamese listeners, tone pairs of CT1-CT3 and CT2-CT5 belonged to the SC type, while CT1-CT6, as well as CT3-CT6, fitted CG.

*Mandarin group*. In a similar vein, the “Mandarin option” exhibited a significant main effect on all Cantonese tones (*p*s < .001, CT3 was excluded from analysis due to 100% assimilation). Then, post-hoc comparisons were conducted again, revealing the following eligible Cantonese-to-Mandarin mappings (*p*s < .001): CT1 to Mandarin Tone 1 (MT1) (99.38%); CT2 to MT2 (98.75%); CT4 to MT3 (82.50%); CT5 to MT2 (85.63%); CT6 to MT1 (84.38%). It was suggested that Mandarin listeners could firmly perceive all Cantonese tones as their native tonal counterparts. Specifically, CT1, CT3, and CT6 were assimilated to MT1, CT2 and CT5 were assimilated to MT2, and CT4 to MT3. Similarly, for the sake of determining the exact assimilation type for tones mapping onto MT1 and MT2, an LMM was computed with “Similarity ratings” and “Cantonese tones” as the dependent variable and fixed effect, respectively; Meanwhile, “Subject” and “Item” were added as random factors. For MT1, there were negligible differences between CT1 and CT3 [*β* = 0.35, *SE* = 0.15, *t* = 2.39, *p* = 0.15], yet a significant discrepancy was detected on CT1-CT6 [*β* = 1.24, *SE* = 0.2, *t* = 6.29, *p* < .001], and CT3-CT6 [*β* = 0.88, *SE* = 0.17, *t* = 5.12, *p* < .01]. For MT2, similarity scores of CT2 and CT5 were comparable to each other [*β* = 0.42, *SE* = 0.12, *t* = 3.69, *p* = 0.06]. In sum, for Mandarin listeners, tone pairs CT1-CT3, and CT2-CT5 fitted the SC, while CT1-CT6 and CT3-CT6 fitted the CG.

Collectively, the assimilation patterns of the Vietnamese and Mandarin groups revealed many commonalities. That is, both had an identical number of TCs, and identified tonal contrasts by height as SC or CG and contrasts by contour as TC, which is closely related to the absence of level tonal contrasts in the Vietnamese and Mandarin systems. According to PAM’s tenets, Vietnamese and Mandarin listeners would encounter greater difficulties with SC and CG pairs relative to TC pairs. In other words, they were more sensitive to tonal contrasts by contour than those by pitch height. However, despite these similarities, the percentages and similarity scores of assimilated tones differed significantly between the two language groups. Relative to their Vietnamese counterparts, Mandarin listeners consistently gave higher percentages and rating scores to the corresponding tones. This suggested that they might perceive Cantonese tones as more similar to their native categories. In order to provide a quantitative assessment of this discrepancy, the fit index and the degree of response diversity (*K′*) were further calculated.

#### Fit index

**Fig 3:**
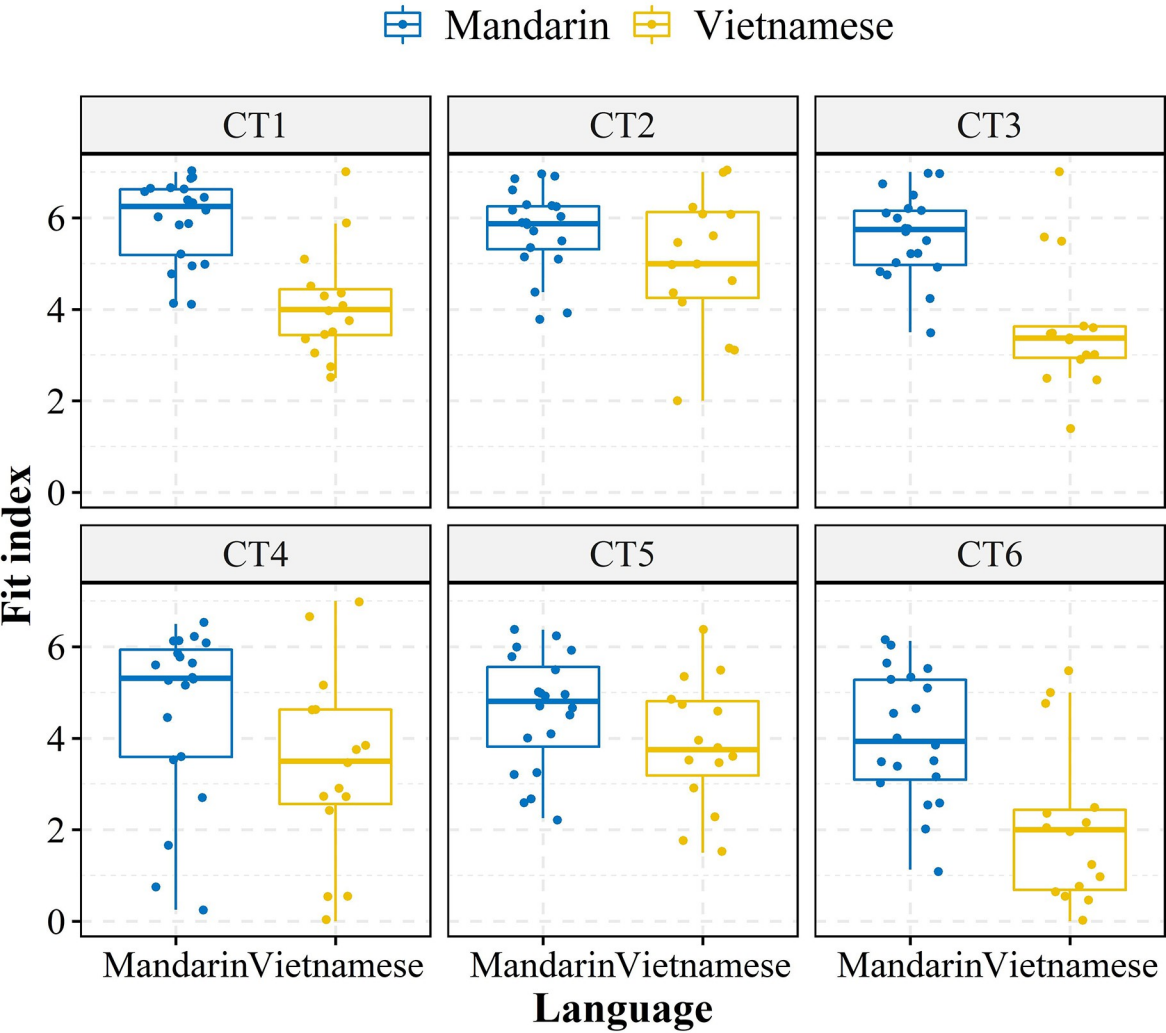
Fit indexes of six Cantonese tones to corresponding assimilated tones in Mandarin and Vietnamese systems. CT = Cantonese tone.

The Vietnamese and Mandarin listeners’ response fit indexes for assimilated tones were submitted to LMM. “Fit index” was treated as the dependent variable, with “Group” and “Cantonese tone” as fixed effects. “Subject” was calculated as the random effect. The visual inspection of Q-Q plots and residuals indicated no obvious deviations from homoskedasticity after the exclusion of extreme data (absolute residuals greater than 2.5) using model-based trimming. Conspicuous distinctions between the two groups in terms of the maximum fit indexes for six tones are displayed in Fig 3. Significant main effects of “Group” [*x*^2^ (1) = 83.69, *p* < .001] and “Cantonese tone” [*x*^2^ (5) = 99.68, *p* < .001] were found, as well as the interaction effect between them [*x*^2^ (5) = 14.23, *p* < .05]. A further post-hoc comparison was conducted to unlock the interaction effect, which revealed that the fix indexes of the assimilated tones in Mandarin were significantly larger than those in Vietnamese across all Cantonese tones (*p*s < .05). This suggests a higher assimilation fit between Mandarin and Cantonese tonal systems. These findings indicate that the perceptual distance between the Cantonese and Mandarin tone systems was smaller than that of the Vietnamese tonal system.

#### Degree of diversity

**Fig 4:**
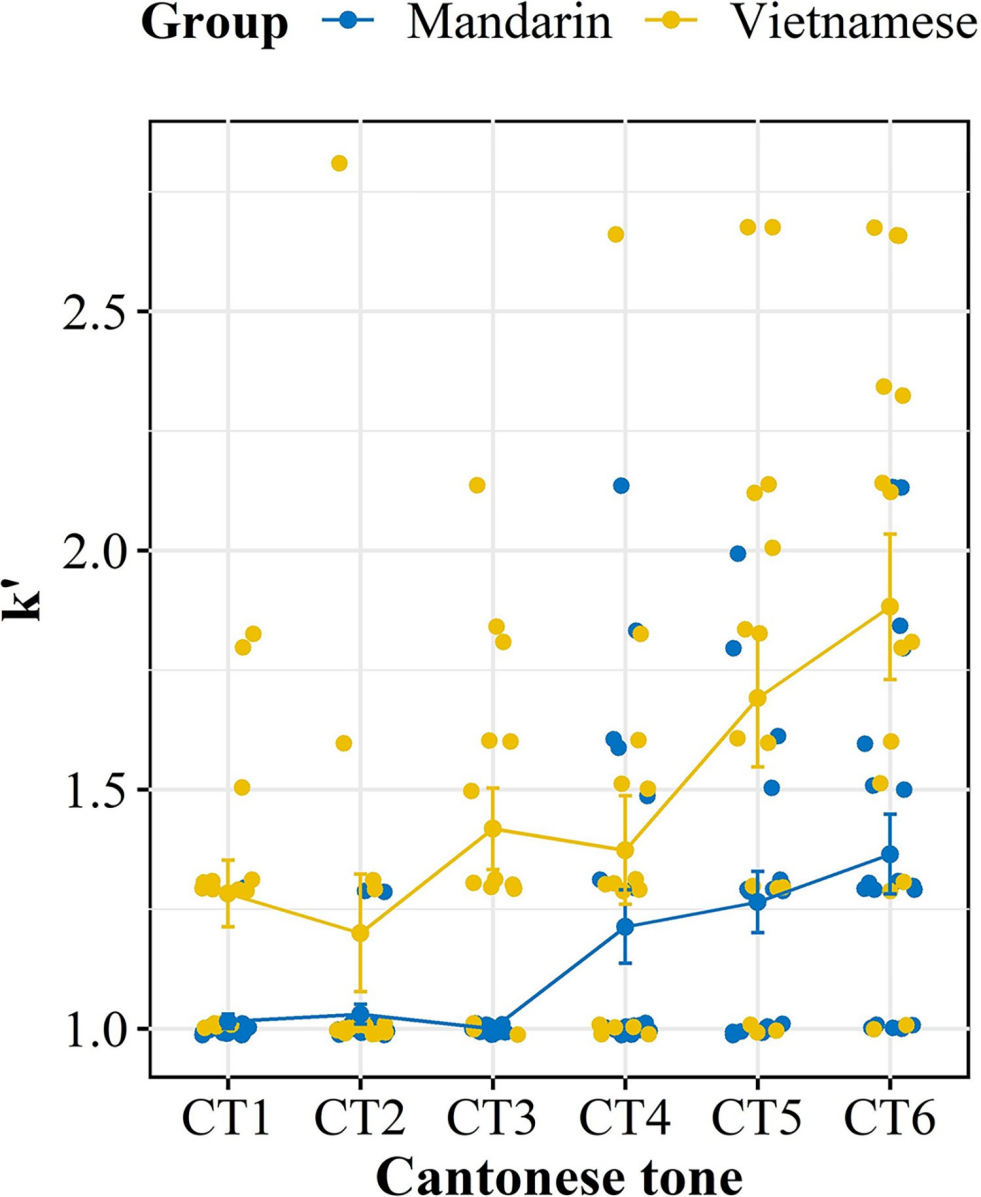
The degree of assimilation diversity (*K*′) of Vietnamese and Mandarin groups for six Cantonese tones. CT = Cantonese tone.

In addition to the fit index, the *K*′ value, which measures the degree of response diversity, was also used to measure how well Cantonese tones were assimilated into the Vietnamese and Mandarin native categories. The *K*′ values of both groups for each Cantonese tone are depicted in Fig 4. Similarly, listeners’ *K′* data were submitted to LMM. “*K′* value” was calculated as the dependent variable, while “Group” and “Cantonese tone” were computed as fixed effects. “Subject” was added as a random factor. There were no obvious deviations from homoskedasticity through the visual inspection of Q-Q plots and plots of residuals after removing extreme data by model-based trimming. Statistical results indicated significant main effects of “Group” [*x*^2^ (1) = 70.30, *p* < .001] and “Cantonese tone” [*x*^2^ (5) = 100.78, *p* < .001], as well as a significant interaction effect of “Group” × “Cantonese tone” [*x*^2^ (5) = 25.06, *p* < .001]. Post-hoc analysis of the interaction effect suggested that the Mandarin group had a significantly lower *K′* than the Vietnamese group for CT3, CT5, and CT6 (*p*s < .01). However, there was no significant difference in *K′* between the groups for CT1 (*p* = 0.21), CT2 (*p* = 0.99) and CT4 (*p* = 0.41). A lower *K′* is indicative of a robust mapping between the L1 and L2 categories. Hence, the significantly lower *K*′ value in the Mandarin group compared to the Vietnamese group might imply that the Mandarin group could more readily and consistently establish mappings between Cantonese tones and their native counterparts.

Combined with the disparities observed in the fit index, it appears that canonical tone languages differ significantly in terms of perceptual similarities despite having similar assimilation patterns (i.e., the number of TC contrasts). Specifically, Cantonese was perceived to be more similar to Mandarin than to Vietnamese, even though the latter has an identical inventory size as Cantonese. Additionally, it was unexpected to find that, despite having a higher tonal density in Vietnamese, this did not result in more TC types for Cantonese tones. The results suggested that high tonal density may not necessarily confer perceptual advantages. On the contrary, since Mandarin was perceived to be closer to the target language than Vietnamese, Mandarin listeners were expected to outperform their Vietnamese counterparts in the discrimination of Cantonese contour pairs (TC types), because they could better deploy their native experience. As for Japanese listeners, it was anticipated that their lack of overt tonal categories might be compensated by their sensitivity to pitch height, as tonal contrasts by height would pose a great challenge to Mandarin and Vietnamese listeners as SC or CG types. These predictions would be tested in the discrimination task below.

### Perceptual discrimination task

Below are the results of three experimental groups (Vietnamese, Mandarin, and Japanese groups) and one control group (Cantonese group) in distinguishing Cantonese lexical tones and pure tones.

#### Overall performance in the discrimination of speech and non-speech tones

**Fig 5:**
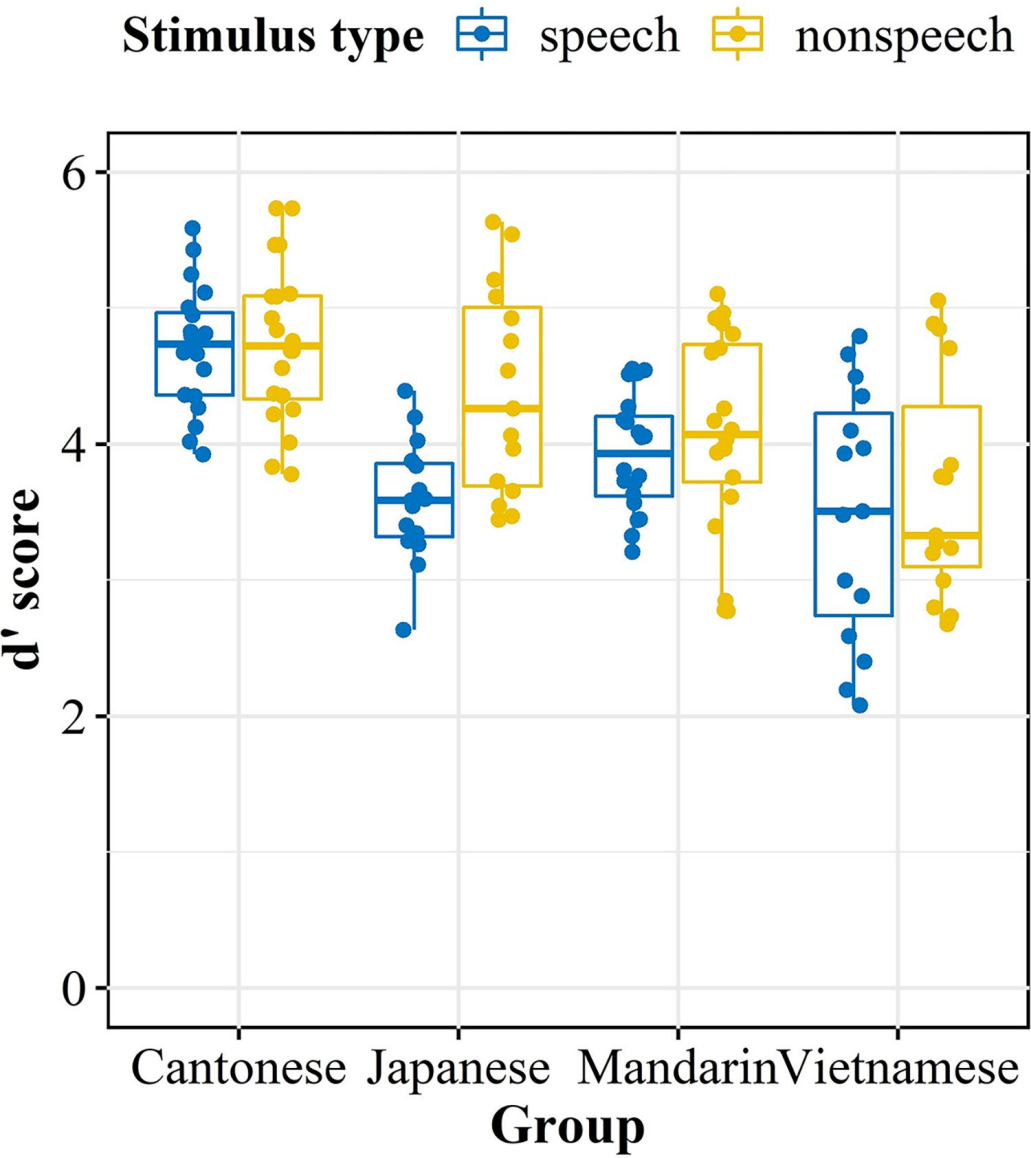
Mean *d*′ scores in tonal discrimination for speech and non-speech types as a function of language group.

In the discrimination task, the Vietnamese, Mandarin, Japanese, and Cantonese control groups achieved mean *d′* scores of 3.5, 3.93, 3.58, and 4.71 for speech tones, and 3.67, 4.08, 4.39, and 4.75 for non-speech tones, respectively. Fig 5 displays the overall performance of the four groups as a function of stimulus type. For statistical analyses, LMMs from the R package *lme4* were implemented. “*d′* score” was calculated as the dependent variable, while “Group” and “Stimulus type (speech vs. non-speech)” were considered as fixed effects. In addition, “Subject” and “Item” were computed as random effects after model comparisons.

Results showed no significant main effect of “Stimulus type” [*x* ^2^ (1) = 1.69, *p* = 0.19]; However, the significant main effect of “Group” [*x* ^2^ (3) = 99.16, *p* < .001], and the interaction effect between them [*x* ^2^ (3) = 10.30, *p* < .05] were observed. Subsequent simple main effect tests were conducted to unlock the interaction effect. First, the effect of “Group” on “Stimulus type” revealed that the Cantonese control group significantly outperformed the three non-native counterparts for speech tones (*p*s < .001). In the case of non-speech pure tones, the Cantonese group still significantly outperformed the Vietnamese and Mandarin groups (*p*s < .01), but performed comparably to Japanese listeners [*β* = 0.33, *SE* = 0.21, *t* = 1.57, *p* = 0.40].

As for the comparisons among the experimental groups, it was found that Mandarin listeners performed significantly better than their Vietnamese counterparts only for the speech type [*β* = 0.41, *SE* = 0.15, *t* = 2.69, *p* < .05], whereas they did not differ in the non-speech context [*β* = 0.39, *SE* = 0.21, *t* = 1.81, *p* = 0.27]. Furthermore, while Vietnamese listeners performed similarly to their Japanese peers in the discrimination of speech tones [*β* = 0.09, *SE* = 0.16, *t* = 0.56, *p* = 0.94], they were significantly less sensitive than the latter in the discrimination of non-speech pure tones [*β* = -0.71, *SE* = 0.23, *t* = -3.16, *p* < .01]. As for the contrast between Mandarin and Japanese listeners, no significant differences in *d′* scores were observed regardless of the stimulus type (*p*s > .05). The findings indicate that, although Vietnamese has a denser tonal system, it does not seem to confer advantages to Vietnamese listeners in the discrimination of both speech and non-speech tones. In addition, unlike Cantonese, Vietnamese, and Mandarin listeners, who performed similarly in speech and non-speech contexts (*p*s > .05), Japanese listeners exhibited a significantly better performance when the tones shifted from speech to non-speech [*β* = 0.80, *SE* = 0.38, *t* = 2.14, *p* < .05], suggesting a domain-specific pitch processing mechanism in Japanese.

#### Discrimination performance for contrast types

**Fig 6:**
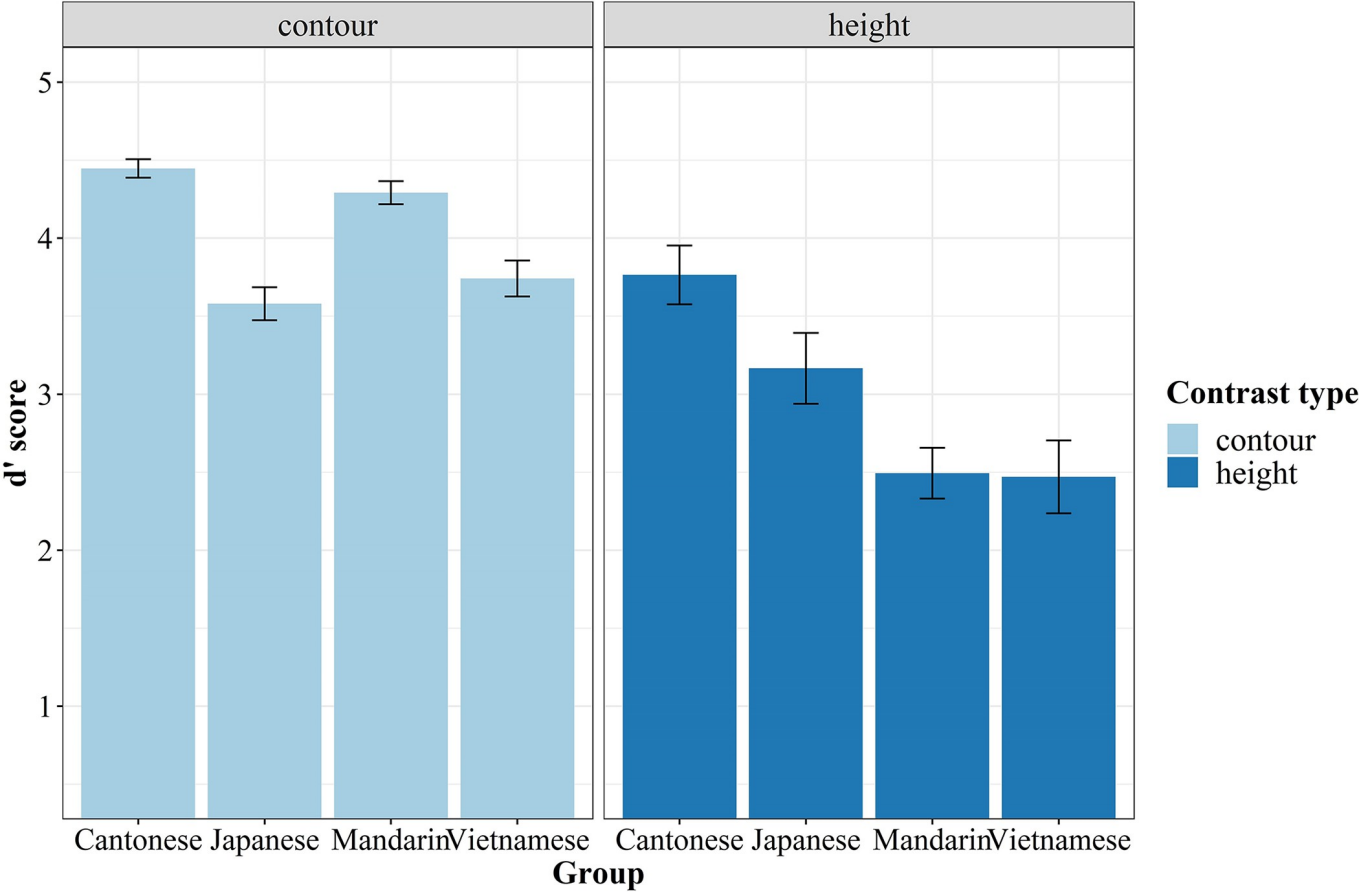
Mean *d′* scores (±SE) for specific contrast types as a function of language group.

In order to explore how listeners’ native prosodic features affected the perception of non-native lexical tones, listeners’ cue weighting was evaluated by comparing their performance in distinguishing specific tonal contrasts (height vs. contour). Fig 6 depicts the discrimination sensitivity to the two contrast types among the four language groups. For the statistical analysis, a new LMM was implemented. The “*d′* score” was counted as the dependent variable, with “Group” and “Contrast type” being computed as fixed effects. In addition, “Subject” with “Syllable (/ji/ and /fu/)” and “Tone pair” with “Syllable” were calculated as random effects.

Both significant main effects of “Group” [*x* ^2^ (3) = 373.33, *p* < .001] and “Contrast type” [*x* ^2^ (1) = 15.42, *p* < .001] were found. Furthermore, their interaction effect was significant [*x* ^2^ (3) = 188.39, *p* < .001]. Simple main effect tests showed that Cantonese native listeners outperformed their three non-native peers for both contrast types (*p*s < .05). In the discrimination of contrasts by contour, the Mandarin group achieved a significantly higher score than both Vietnamese and Japanese groups (*p*s < .001); However, there was no significant difference between the Vietnamese and Japanese groups (*p* = 0.14). Conversely, when distinguishing tone pairs differing in pitch height, Japanese listeners significantly outperformed the two canonical tonal groups (*p*s < .001), whereas there were negligible differences between the Vietnamese and Mandarin groups (*p* = 0.99).

Regarding contour versus height performance within each group, the results showed distinct perceptual patterns. Analogous to the Cantonese control group, Japanese listeners performed equally well for both types of tonal pairs (Cantonese: *p* = 0.30; Japanese: *p* = 0.79). On the one hand, Japanese pitch-accent patterns would lend benefits to their discrimination of pitch height [19, 40, 56]. On the other hand, tone pairs contrasted by contour are inherently more distinctive in acoustics, which would contribute to high discriminability regardless of listeners [2]. In contrast, both Mandarin and Vietnamese listeners performed significantly better for contour pairs than height ones (*p*s < .01), possibly due to the lack of minimal contrasts between level tones in their native tonal systems. Moreover, their slanted advantages for contour pairs also corroborated the PAM’s protocol that TC pairs would be easier to discriminate than SC or CG pairs. Since Vietnamese and Mandarin groups were identical in the assimilation patterns (the number of TC, SC, or CG types), significant differences among them could not be addressed by PAM; Thus, listeners’ specific discrimination performance on the 15 tonal pairs (all possible combinations) was not analyzed and reported in this study.

## Discussion

Results of the assimilation and discrimination tasks revealed that L1 tonal density could modify naïve listeners’ perception of non-native tones. Furthermore, speech and non-speech tones were processed similarly by canonical tone language groups but differently by the pitch-accent language group, indicating a language-specific mechanism in pitch processing across domains. The preceding findings will be discussed from the following two aspects.

### The effect of L1 tonal density

It was revealed that the size of the L1 tonal inventory would affect listeners’ perceptual similarity of Cantonese tones, which further influenced their discrimination of tonal contrasts. Regarding the first question, the results of the perceptual assimilation task found that, compared to their Vietnamese peers, Mandarin listeners exhibited steadier assimilation patterns with higher percentages and similarity ratings. Moreover, a significantly higher fit index and lower response diversity (*K′*) were observed in the Mandarin group, indicating that the assimilation consistency of each Cantonese tone category mapped onto the Mandarin category was higher than that of the Vietnamese group. All these findings suggest that L1 tonal density could affect listeners’ perceptual similarity between the target language and their mother tongue [50]. Surprisingly, a denser L1 tone inventory would lead to a larger perceptual gap between the two prosodic inventories. On the one hand, it is possibly due to denser tonal listeners’ undue sensitivity to the subtle L1-L2 phonetic differences, yielding a decrease in L1-L2 perceptual similarity [25]. Another reason accounting for this could stem from a cognitive perspective. A larger L1 tonal inventory may impose a higher working memory load on its listeners during tonal assimilation. In this study, Vietnamese listeners may have been distracted by irrelevant native tonal categories in pitch processing because they needed to make more comparisons than their Mandarin counterparts before determining assimilation categories. This speculation is further supported by feedback from both Vietnamese and Mandarin listeners regarding the experimental difficulty.

Regarding the second question, discrimination results demonstrated the superiority of Mandarin listeners over their Vietnamese peers, yet there were comparable performances between the Mandarin and Japanese groups, as well as between the Japanese and Vietnamese groups. Additionally, Vietnamese and Mandarin listeners performed significantly better for tonal pairs contrasted by contour than those contrasted by height, whereas Japanese listeners exhibited considerably higher sensitivity than the other two groups in discriminating contrasts by height. These results suggest that listeners’ L1 perceptual cues could transfer to the perception of a non-native language [2, 18]. This being the case, listeners of canonical tone languages attended more to pitch contour, while those from pitch-accent languages paid more attention to pitch height, in conformity with previous studies [19, 56]. Indeed, pitch-accent languages, though generally classified as tone languages, are phonetically more similar to non-tone languages [43].

Aside from the group discrepancies between canonical tone languages and the pitch-accent language, performance within canonical tone languages (Vietnamese vs. Mandarin) could be much more noteworthy. Despite having a larger tone inventory, Vietnamese listeners performed worse than their Mandarin counterparts, who had fewer tonal categories. The results seem to suggest that a higher L1 tonal density would not assist and may even hinder listeners from distinguishing non-native tonal contrasts [6, 11]. One explanation could be related to the L1-L2 perceptual similarity reflected by the fit index and *K*′ value. Mandarin listeners more consistently assimilated Cantonese tones into their native system, allowing novice listeners to benefit from their native tonal system by locating their native counterparts to distinguish non-native tones. It echoes the fact that Mandarin listeners outperformed Vietnamese solely for contour pairs, which belonged to TC types. It was also found that listeners could better deploy their native phonemic knowledge only when one tone pair was assimilated into two categories [57]. In summary, the results might suggest a positive association between L1-L2 perceptual similarity and discrimination sensitivity in the case of TC types. However, our results contradict the L2LP’s scenario by demonstrating that a larger tone inventory might not guarantee more TC types when compared to a sparser system.

Regarding previous studies on non-native tone perception, the findings partially dispute the claims made by [9, 12] that a denser tonal system has a facilitative effect. However, the reliability of these studies is limited by some factors. For instance, the study [9] did not compare non-native listeners with different tonal backgrounds when they tested the perception of a new language. They only examined the relationship between the complexity of the native language and the target language since there was only one non-native tone group. Moreover, it also bears some uncontrolled factors, such as musical experience, which might influence the results. Furthermore, the previous study [12] tested listeners’ categorical perception of native tones in both Mandarin and Cantonese, which is essentially different from non-native tone perception behaviorally and physiologically [3, 58].

The results support the findings of [6, 11], who found that listeners from canonical tone languages did not outperform listeners from pitch-accent languages with limited pitch variations. Additionally, the negative role of a denser L1 inventory was upheld by rich research at the segmental level. One study [25] compared French and Spanish vowel inventories and advocated that speakers of languages with larger inventories would perceive the same sounds as less similar than speakers with smaller inventories, aligning with the view of separation between language-specific perceptual space and universal acoustic space. A similar result was shown by [23], who argued that Spanish listeners outperformed their English peers in the discrimination of Portuguese vowel contrasts. However, it should be acknowledged that besides the inventory of tones, voice quality might also contribute to Vietnamese listeners’ lower performance in Cantonese tone perception. A denser tonal system is usually accompanied by voice quality along with pitch to distinguish tones, as seen in languages like Hmong [6]. In contrast to Mandarin and Cantonese, Vietnamese relies heavily on phonation in distinguishing tones, which may distract listeners from pitch processing.

### The effect of L1 tonal typology

Aside from the number of tones, a finer tonal typology in L1 (canonical tone vs. pitch accent) has been shown to affect non-native tone perception. In response to the third question, both lexical and analogous pure tones were adopted in the discrimination task. Results observed an asymmetric pattern in the Japanese group across different stimulus types. Specifically, the three groups of canonical tone languages, including Cantonese native listeners, performed equally well for both linguistic and non-linguistic tones. In contrast, for the pitch-accent language group, Japanese listeners improved their performance significantly when the stimulus type changed from lexical to pure tones. It suggested that pitch processing mechanisms across domains might be language-specific, depending on the tonal typology. Specifically, listeners of canonical tone languages appeared to utilize a domain-general mechanism when perceiving speech and non-speech pitch contours, in line with previous studies [30, 31, 34, 36]. On the other hand, listeners of pitch-accent languages may rely on different mechanisms for perceiving lexical tones and non-speech analogues. It also suggested that native experience had a specific linguistic effect rather than a general effect on non-native tone perception. The findings observed in Japanese listeners are in line with the findings of [15, 35], which focused on infants and adults from non-tone language backgrounds.

One explanation for this asymmetry could be related to Japanese-specific prosodic features. As introduced before, although Japanese bears pitch variations at the word level, it lacks overt tones on monosyllables [1]; Moreover, this limited “tonal” experience only occurs for a few words [11]. However, Japanese listeners’ phonological restrictions at the lexical level would be removed when they perceived pure tones, which were analogous to their non-tone language counterparts [35]. This is echoed by the fact that canonical tone language listeners would process segmental information and tone integrally while non-tone and pitch-accent language listeners would not [9, 19]. In general, despite the similarity in pitch functions, canonical tone language and pitch-accent language could diverge significantly in speech and non-speech processing mechanisms due to the effect of tonal typology. In future studies, non-native tone perception could be examined in the field of electrophysiology to validate behavioral findings and gain insights into the neural mechanism of pitch processing.

Admittedly, the current study has several limitations. Firstly, each language group contained a limited sample size, which might reduce the statistical power. In addition, the Vietnamese group’s native experience regarding voice quality might affect the results; Hence, more tone language groups could be included in future research to verify the current findings. For instance, tone languages that have more complex systems or Bantu languages that lack contour tones could be investigated in future studies. Furthermore, the current study investigated non-native tone perception only in citation form. More complex forms, such as dissyllables and sentence contexts, could be considered in future studies to provide a better understanding of this issue from a more dynamic perspective.

## Conclusions

The present study investigated the effects of L1 tonal density and typology on the perception of Cantonese lexical and pure tones in three groups of tone-language speakers with different total inventories. It was found that Mandarin listeners significantly outperformed their Vietnamese counterparts in the discrimination of Cantonese tonal contrasts, suggesting that a larger tone inventory did not benefit listeners, and could even exert a detrimental effect on the perception of a novel tone language. Besides, an asymmetric pattern was observed only in the Japanese group, who performed significantly better for pure tones than speech ones, indicating a domain-specific processing mechanism for pitch-accent languages. Taken together, both L1 tonal size and L1 tonal typology would modulate non-native tone perception.
